# Molecular characterization of non-*Cryptococcus* yeast communities isolated from *Eucalyptus* trees

**DOI:** 10.22034/CMM.2024.345184.1500

**Published:** 2024-03-29

**Authors:** Hasti Nouraei, Fatemeh Gharechahi, Zahra Zareshahrabadi, Kamiar Zomorodian, Alireza Gharavi, Hossein Khodadadi, Saham Ansari, Neda Amirzadeh, Keyvan Pakshir

**Affiliations:** 1 Department of Parasitology and Mycology, School of Medicine, Shiraz University of Medical Sciences, Shiraz, Iran; 2 Basic Sciences in Infectious Diseases Research Center, Shiraz University of Medical Sciences, Shiraz, Iran; 3 Department of Parasitology and Mycology, School of Medicine, Shahid Beheshti University of Medical Sciences, Tehran, Iran

**Keywords:** *Eucalyptus*, Molecular identification, Natural flora, Yeast

## Abstract

**Background and Purpose:**

Plants are crucial habitats for fungus communities as they provide an appropriate physical environment for the growth and reproduction of the yeast microbiome. Varieties of pathogenic and non-pathogenic yeast could be found in *Eucalyptus* trees. Although *Cryptococcus* species are the most common pathogenic yeasts associated with *Eucalyptus* trees, other yeasts also grow on trees and are critical to human health. This study aimed to identify the yeast species associated with *Eucalyptus* trees.

**Materials and Methods:**

In total, 107 yeast species were collected from *Eucalyptus* trees and subsequently identified through both molecular and traditional techniques. Genomic DNA extraction was performed using the boiling method. The internal transcribed spacer region of the ribosomal DNA was amplified utilizing the polymerase chain reaction (PCR) technique, followed by the purification and sequencing of the PCR products to identify the isolates.

**Results:**

Yeast strains belonged to 12 genera and 26 species of both the *Ascomycete* and *Basidiomycete* phyla. The most frequent species were *Rhodotorula mucilaginosa* (24.2%), *Candida tropicalis* (15%), *Candida guilliermondii* (11.2%), and *Aureobasidium pullulans* (10.2%).

**Conclusion:**

In this study, most of the yeast isolates, such as *Candida* and *Trichosporon*, were important to human health. *Eucalyptus* trees, as part of the natural flora, could be considered an environmental reservoir for yeasts, in which they can survive, disperse to the surrounding environment, and become a potential infectious source affecting public health

## Introduction

The term 'phyllosphere' pertains to the external parts of plants above the ground, such as leaves, stems, buds, flowers, and fruits. These areas provide living spaces for a variety of microorganisms. The composition of microbial populations on these plant components can vary, including those that reside on the surface (epiphytes) and those that inhabit inside (endophytes), based on the nature of the host plant itself. Some microorganisms face challenges in adapting and thriving within the phyllosphere, whereas epiphytic species manage to survive, grow, and reproduce on the exterior of plants. The phyllosphere hosts an extensive array of microbes, encompassing bacteria, archaea, yeasts, and filamentous fungi. 

*Eucalyptus* trees are widely distributed in some parts of the world and have also been introduced as ornamental trees in many countries. Furthermore, *Eucalyptus* trees have been used for therapeutic purposes, such as fighting malaria, in many places. These trees are habitats for living pathogenic and non-pathogenic yeasts. The micro-environmental conditions on the tree tissue surfaces affect the fungal establishment and provide a suitable physical environment for their growth and reproduction. 

The growth of the epiphytic yeast population in the trees depends strongly on nutrient materials, water, and protection [ 1 , 2 ]. Yeasts and yeast-like fungi have been found in decomposing wood inside tree trunk hollows, barks, leaves, and other plant components [ 3 ]. Although *Cryptococcus* species are the most common pathogenic yeast associated with *Eucalyptus* trees, there are a variety of other yeasts that grow on trees and are beneficial to human health. *Candida*, *Cryptococcus*, *Debaryomyces*, *Hansenula*, and *Pichia* spp. were found to be the most common white yeasts, while red yeasts, mostly from the genera *Rhodotorula* and *Sporobolomyces*, were found in smaller numbers [ 2 , 4 - 6 ]. Some of the related yeasts have the potential to transmit to humans and cause disease. 

The identification of yeast fungi at the species level is critical in several fields of research for various purposes, including finding appropriate treatments, elucidating epidemics, and understanding transmission pathways in health and agriculture [ 7 , 8 ]. The rise in the occurrence of these diseases is linked to treatments involving immunosuppressants, corticosteroids, and prolonged antibiotic use, particularly after organ transplants or in cases of severe metabolic, hematological, or immune disorders [ 9 , 10 ]. Recently, several researchers have reported that *Ascomycetous* yeast genera, including *Debaryomyces*, *Hanseniaspora*, *Hansenula*, *Kluyveromyces*, *Pichia*, and *Succharomyces*, have killing activities [ 11 ]. Killer yeasts can produce and excrete extracellular enzymes that are toxic to some sensitive microorganisms, including bacteria (such as *actinomycetes*) and filamentous fungi. The yeast-killer phenomenon may also play a substantive role in stabilizing the ecosystem [ 12 ].

Although several studies continue to focus on the microbial diversity in *Eucalyptus* [ 13 - 15 ], there are no reports of the yeast community associated with *Eucalyptus* trees in Iran. In our previous study on molecular epidemiology, environmental *Cryptococcus* spp. were isolated [ 16 ]; however, the present study aims to evaluate the diversity of other non-*Cryptococcus* yeasts and yeast-like organisms associated with Eucalyptus trees in Shiraz, Iran.

## Materials and Methods

### Sampling and Sample Processing

For the isolation of non-*Cryptococcus* spp., 146 yeast samples from our previous study were subcultured on Sabouraud dextrose agar ([SDA], Merck, Germany) with pH=5.6±0.2, supplemented with chloramphenicol (200 mg/L) [ 16 ], incubated at 32°C for 48 h, and inspected daily for growth.

### Conventional Identification Methods

Primary identification of yeast isolates was conducted through the micro-morphological examination of vegetative yeast cell forms (round, ovoid, or rectangular) observed in SDA via teased mount preparations. Additionally, the growth patterns on Cornmeal-Tween-80 agar ([CMA], Merck, Germany) were assessed to detect the presence of pseudohyphae and chlamydoconidia. The ability to produce germ tubes in serum and the distinct colony pigmentation on CHROMagar-*Candida* media (Paris, France) were also crucial criteria for identification.

### Molecular Identification Method

### DNA Extraction

The procedure was based on the boiling method outlined by Makimura et al. [ 17 ]. To summarize, a few fresh yeast colonies were initially suspended in a lysis buffer containing 100 mM Tris-HCl, 0.5% SDS, and 30 mM EDTA, followed by a gentle 15-min boiling. Subsequently, 2.5 M potassium acetate was added, and the mixture was incubated for 1 h. After this, it was spun down at 12,000 rpm for 5 min, and the supernatant, the clear layer above the pellet, was transferred to a fresh tube. This extract, containing yeast DNA, was subjected to dual ethanol washes, allowed to air dry, and then redissolved in 50 μL of distilled water for further analysis via polymerase chain reaction (PCR). The quality and quantity of the DNA were assessed by its A260/A280 absorbance ratio and nano-drop measurements. The extracted DNA was then stored at -20°C for future use.

### Amplification and DNA Sequence Analysis

### Amplification

The internal transcribed spacer1 (ITS1)-5.8S-ITS2 of the ribosomal DNA region was chosen as the target for amplification by ITS1 (5’-TCC GTA GGT GAA CCT GCG G-3’) and ITS4 (5’-TCC TCC GCT TAT TGA TAT GC-3’) primers [ 18 ]. The amplification process was performed in a total reaction volume of 50 μl, containing 5 µl PCR buffer, 0.5 µl dNTP, 0.5 µl Taq, 0.5 µl ITS1 and ITS4, 2 µl genomic DNA, 1.5 µl mgcl2, and 39.5 µl dH2O. 

**Figure 1:**
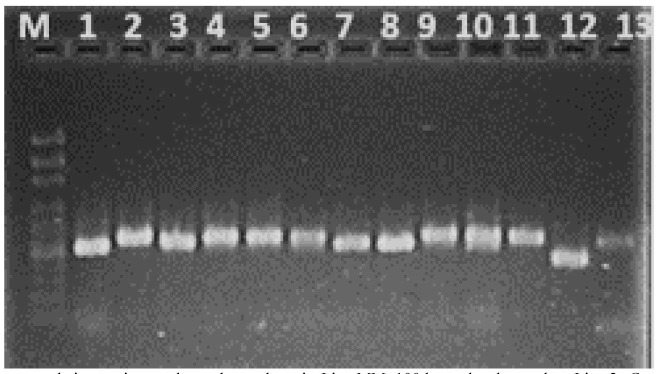
Results of polymerase chain reaction products electrophoresis. Line MM: 100 bp molecular marker; Line 2: *Candida guilliermondii*; Lines 1, 3, and 12: *Rhodotorula sloofia*; Lines 4: *Aureobasidium pullulans*; Line 5, 6, 7, 10, 11, and 13: *Rhodotorula mucilaginosa*; Lines 8 and 9: *Trichosporon asahii*.

The PCR protocol was established with the initial denaturation step at 95°C for 5 min, followed by 35 cycles that included denaturation at 94°C for 30 sec, annealing at 62°C for 30 sec, and extension at 72°C for 40 sec. This was concluded with a final extension at 72°C lasting for 5 min. The PCR product was visualized by electrophoresis on 1.2% agarose gel and stained with ethidium bromide. The stained gels were photographed using the UVITEC system (Figure 1).

### Sequencing

The PCR products were purified and sequenced (Bioneer Company, South Korea). After configuring the sequences to a FASTA format, the sequence results were analyzed, and species were identified by searching databases after depositing them to the online BLAST system at the website of the National Center for Biotechnology Information (http://www.ncbi.nlm.nih.gov) to confirm the identified strains.

### Ethical Approval

This project was in accordance with the ethical principles and the national norms and standards for conducting medical research in Iran, and it has been approved by the Research Ethics Committee of Shiraz University of Medical Sciences, Shiraz, Iran (IR. SUMS.REC.1393.7221).

## Results

### Morphological and Sequence Results

**Figure 2:**
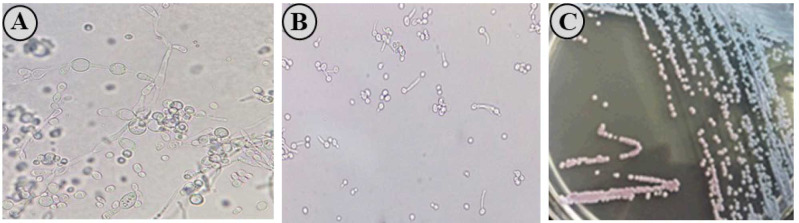
A) Chlamydoconidia test, B) Germ tubes test, and C) CHROMagar-*Candida* media

The results of 107 various yeast species and yeast-like fungi based on conventional and morphological characteristics are presented in Table 1. Based on the findings, 27 *Candida* species ([spp.], 25.2%) presented pseudohyphae, and 2 strains (1.8%) were phenotypically identified as *Candida albicans* (*C. albicans*) spp. complex on the basis of a positive germ tube test and also formed *Chlamydoconidia* in CMA with Tween 80. In addition, among all strains, *C. albicans*, *C. tropicalis*, and *C. glabrata* presented green, blue, and purple colony colors, respectively, while the other yeast species could not produce any color and remained white (Figure 2). 

**Table 1:** Identification of yeast species by DNA sequencing of the internal transcribed spacer region

N (%)	Yeast species	N (%)	Yeast species
2 (1.8)	*Torulaspora delbrueckii*	26 (24.2)	*Rhodotorula mucilaginosa*
2 (1.8)	*Candida magnoliae*	16 (15.0)	*Candida tropicalis*
1 (0.9)	*Rhodosporidium babjeva*	12 (11.2)	*Candida guilliermondii*
1 (0.9)	*Rhodospridium lusitaniae*	11 (10.2)	*Aureobasidium pullulans*
1 (0.9)	*Monilliela pollinis*	5 (4.7)	*Trichosporon asahi*
1 (0.9)	*Candida glabrata*	5 (4.7)	*Hanseniaspora uvarum*
1 (0.9)	*Candida zeylanoides*	4 (3.7)	*Metschinkowia viticola*
1 (0.9)	*Pichia kudriavzevii*	4 (3.7)	*Pichia fermentans*
1 (0.9)	*Pichia kluyveri*	3 (2.8)	*Candida apicola*
1 (0.9)	*Candida* spp.	3 (2.8)	*Rhodotorula sloofiae*
1 (0.9)	*Kodamaea ohmeri*	2 (1.8)	*Candida albicans*
1 (0.9)	Yeast unknown	2 (1.8)	*Meyerozyma carribica*
107 (100)			Total

The identified yeasts belonged to 12 genera and 22 species of both *Ascomycetous* and *Basidiomycetous* genera. Among the 107 isolated yeast strains, the most frequent species were *Rhodotorula mucilaginosa* ([26 24.2%]), *C. tropicalis* ([16 15.0%]), *C. guilliermondii* ([12 11.2%]), and *Aureobasidium pullulans* (*A. pullulans*], [11 10.2%]). Other genera were identified as *Trichosporon*, *Hanseniaspora*, *Metschinekowia*, *Torulaspora*, and other species of *Candida*, which are presented in Table 1. Moreover, *Rhodosporidium* and *Rhodotorula* spp. were also identified as red yeasts, and *Aureobasidium* and *Monilliela* were identified as black yeasts in this study.

## Discussion

Nowadays, people spend most of their time outdoors; therefore, they are likely to be exposed to the effects of microorganisms in the environment. *Eucalyptus* plantations provide a low-cost and renewable source of raw materials. However, little is known about their microbiology. The present study characterized the natural occurrence, prevalence, and colonization of pathogenic and non-pathogenic yeasts associated with the natural flora of *Eucalyptus* trees in Iran. *Eucalyptus* trees are the main habitat for *Cryptococcus* spp., especially *Cryptococcus gattii* and *Cryptococcus neoformans*; however, many studies have shown that other species of yeasts have also been isolated from *Eucalyptus*, which is thus considered a possible source of fungal infections affecting humans and animals [ 4 , 19 ]. 

Although many fungi tend to habituate *Eucalyptus* trees, maybe due to the attractive materials existing on these trees, the main source of contamination of leaves and trunks is bird droppings. The cloaca and intestinal tracts of birds contain many yeast microbiomes, and guano is the main source of contamination in trees [ 20 ]. In the current study, yeast communities found in *Eucalyptus* trees belonged to the *Ascomycete* and *Basidiomycete* phyla, which was similar to the findings of other studies on different trees [ 21 - 23 ]. 

In this study, conventional methods and molecular ITS-sequencing techniques were used for the primary and exact identifications of yeast strains, respectively. All *C. albicans* spp. exhibited similar results using conventional and molecular methods. In accordance with the results of the present study, most of the identified strains, such as *Rhodotorula* spp., *Candida* spp., *Trichosporon*, and *Aureobasidium*, are important opportunistic pathogens for humans that can cause fatal diseases and hazards, especially in immunosuppressed patients.

Moubasher et al., in their previous investigation, focused on fungi on surfaces of citrus and grapevine leaves in a one-year experiment in Egypt and showed that *Cladosporium* and *Alternaria* genera were predominant on citrus and grapevine leaves, respectively [ 24 ]. *Rhodotorula* was the most frequent isolate from *Eucalyptus* trees in the present study. This genus is a common saprophytic yeast that can be found in a variety of environments. The isolation of this fungus from various environments has been described by several previous studies [ 25 - 27 ]. 

*Rhodotorula* spp. is also responsible for many fungal diseases. According to the literature, fungemia is a common *Rhodotorula* spp. infection that usually affects young people. Malignancy, chemotherapy, recent antibiotic treatment, total parenteral nutrition, neutropenia, transplantation, and AIDS were all found to be significant risk factors in *Rhodotorula* fungemia patients [ 28 ]. *C. tropicalis*, the second most frequent yeast in the present investigation, was another important opportunist fungus. *C. tropicalis* is highly abundant in Asia-Pacific and Latin America (14.9%). 

While *C. albicans* remains the most common culprit in candidiasis cases, there is a growing trend of other *Candida* spp., such as *C. tropicalis*, being identified as responsible for invasive *Candida* infections [ 29 ]. *C. tropicalis* is a species of yeast that is commonly found in various natural and human-associated environments and has been obtained from seawater, sea sediments, mudflats, marine fish intestines, mangrove plants, marine algae, and shrimps. This yeast thrives in warm and moist areas and is frequently isolated from soil, water, and plants, indicating its widespread presence in the environment. The adaptability of *C. tropicalis* to diverse environments underscores its ecological versatility and its significance in both environmental and clinical contexts [ 30 ]. Fluconazole and voriconazole resistance, particularly fluconazole resistance, develop more frequently in clinical isolates of *C. tropicalis*, compared to the clinical isolates of *C. albicans*, with rates ranging from 5% to 36% [ 29 , 31 ]. *C. tropicalis* infections in the bloodstream are associated with substantial death rates ranging from 41% to 61% [ 32 , 33 ]. 

*Meyerozyma* (*Candida*) *guilliermondii*, a species ubiquitous in nature and considered an opportunistic human pathogen, accounted for approximately 11.2% of all yeast isolates in this study. In the last decades, less frequent non-*albicans Candida* spp., such as *C. guilliermondii*, have been reported as emerging agents of bloodstream infections with high rates of associated mortality in patients with cancer [ 34 ]. Another yeast isolated in the present study was *A. pullulans* (12% from *Eucalyptus* trees). It is noteworthy that invasive infection with *A. pullulans*, as dematiaceous fungi, has been previously reported, with all reported cases occurring in patients with immunodeficiency conditions, such as cancer, autoimmune diseases, and organ transplants, as well as patients with significant hospital exposure [ 35 , 36 ]. 

Melanin is a virulence factor for these fungi, which localizes to the cell wall and plays a role in the survival of the organism and the pathogenesis of disease. Melanin causes resistance to immune system processes and can protect fungal cells against lysis and phagocytosis. Melanin can also bind hydrolytic drugs, such as some antifungal agents, and thus obstruct their access to the cell membrane [ 37 , 38 ]. 

*Trichosporon asahi* was one of the frequent genera isolated during this study. This genus was also isolated from *Pinus* trees and pigeon droppings in India and Iran, respectively [ 3 , 20 ]. Species of *Trichosporon* are regarded as significant contributors to nosocomial infections [ 39 ]. They are often ranked as the second or third most frequent non-*Candida* yeast infections leading to invasive diseases, particularly in patients suffering from hematological cancer [ 40 ]. In the present study, other uncommon yeast and yeast-like species associated with *Eucalyptus* trees were also isolated, but these species were less frequently responsible for fungal infection. 

In the present study, *Kodamaea ohmeri* was also isolated from *Eucalyptus* trees. In a systematic review, Loannou et al. reported that *K. ohmeri*, recognized as a rare yeast pathogen, can be isolated from environmental sources and may cause life-threatening infections in humans [ 41 ]. Moreover, according to the findings of the present study, *Pichia* (5.5%) and *Hanseniaspora* (4.7) genera were killer yeasts isolated from *Eucalyptus* trees. It is postulated that the existence of killer yeast strains can be used to biologically control the growth of pathogenic microorganisms in *Eucalyptus* trees. In a previous study that was carried out in Argentina, *Pichia* and *Wickerhamomyces* exhibited a significant inhibitory effect against *Penicillium digitatum* in assays using wounded lemons [ 42 ]. Therefore, based on the findings of the present study, *Eucalyptus* trees could be considered an important reservoir for serious pathogenic yeasts with a potential to be transmitted to humans.

## Conclusion

In this study, 107 yeast species associated with *Eucalyptus* trees were identified, comprising both pathogenic and non-pathogenic types. These yeasts pose a threat of serious fungal infections, particularly in immunocompromised individuals. The existence of *Eucalyptus* trees, acting as fungal hosts and carriers, near rest homes and hospitals represents a potential hazard for these vulnerable groups. The strains examined in this study reflect the diversity of fungi found in the contaminated *Eucalyptus* trees of Shiraz. 

In conclusion, there is a need for more environmental studies focusing on extensive sampling from temperate and other climatic zones that were not explored in the present study. Such studies might reveal new yeast genera or species, some of which may have pathogenic potential.
